# Cyclin-Dependent Kinase 5 Inhibitor Butyrolactone I Elicits a Partial Agonist Activity of Peroxisome Proliferator-Activated Receptor γ

**DOI:** 10.3390/biom10020275

**Published:** 2020-02-11

**Authors:** Sungjin Ahn, Dong Man Jang, Sung Chul Park, Seungchan An, Jongheon Shin, Byung Woo Han, Minsoo Noh

**Affiliations:** 1 Natural Products Research Institute, College of Pharmacy, Seoul National University, 1 Gwanak-ro, Gwanak-gu, Seoul 08826, Korea; sungjinahn@snu.ac.kr (S.A.); sungchulpark@snu.ac.kr (S.C.P.); shinj@snu.ac.kr (J.S.); 2 Research Institute of Pharmaceutical Sciences, College of Pharmacy, Seoul National University, 1 Gwanak-ro, Gwanak-gu, Seoul 08826, Korea; jdm721@snu.ac.kr

**Keywords:** butyrolactone I, PPARγ partial agonism, CDK inhibitor, human bone marrow mesenchymal stem cells, polypharmacology

## Abstract

Adiponectin is an adipocyte-derived cytokine having an insulin-sensitizing activity. During the phenotypic screening of secondary metabolites derived from the marine fungus *Aspergillus terreus*, a poly cyclin-dependent kinase (CDK) inhibitor butyrolactone I affecting CDK1 and CDK5 was discovered as a potent adiponectin production-enhancing compound in the adipogenesis model of human bone marrow-derived mesenchymal stem cells (hBM-MSCs). CDK5 inhibitors exhibit insulin-sensitizing activities by suppressing the phosphorylation of peroxisome proliferator-activated receptor γ (PPARγ). However, the adiponectin production-enhancing activities of butyrolactone I have not been correlated with the potency of CDK5 inhibitor activities. In a target identification study, butyrolactone I was found to directly bind to PPARγ. In the crystal structure of the human PPARγ, the ligand-binding domain (LBD) in complex with butyrolactone I interacted with the amino acid residues located in the hydrophobic binding pockets of the PPARγ LBD, which is a typical binding mode of the PPARγ partial agonists. Therefore, the adiponectin production-enhancing effect of butyrolactone I was mediated by its polypharmacological dual modulator activities as both a CDK5 inhibitor and a PPARγ partial agonist.

## 1. Introduction

Phenotype-based drug screening is extensively used in drug discovery as an alternative approach to target-based screening methods [1]. A phenotype-based screening approach is advantageous for chronic diseases associated with diverse etiologies, such as atherosclerosis, obesity, and diabetes. The adipogenesis model of human bone marrow mesenchymal stem cells (hBM-MSCs) has been used for phenotypic screening of metabolic diseases by measuring adiponectin [2,3]. Adiponectin is a major adipocyte-secreting cytokine that regulates various metabolic functions, such as glucose homeostasis and fatty acid oxidation [4]. Notably, the serum adiponectin levels are relatively decreased in patients with obesity, insulin resistance, cardiovascular disease, and hypertension as compared to those in the healthy population [5,6]. In particular, for obesity-related cancers, such as endometrial cancer, leukemia, gastric cancer, and colon cancer, the levels of circulating adiponectin are lower [7,8,9,10,11]. It has been reported that treatment with recombinant adiponectin increases insulin sensitivity and reduces ectopic lipid formation by promoting fat accumulation in the normal adipose tissues [12,13,14]. Adiponectin has anti-inflammatory, anti-atherosclerotic, and cardioprotective effects [15]. In this regard, compounds that enhance adiponectin biosynthesis have been suggested as new therapeutics for various metabolic diseases and cancers. In fact, many pharmacological drugs enhance adiponectin biosynthesis during adipogenesis in hBM-MSCs [16]. Anti-diabetic peroxisome proliferator-activated receptor γ (PPARγ) agonists, such as troglitazone and pioglitazone, as well as sulfonylureas, such as glibenclamide, significantly enhance adiponectin production during adipogenesis in hBM-MSCs [17,18]. Although a direct molecular target has not been identified yet, aspirin upregulates adiponectin production in the differentiated adipocytes in a concentration-dependent manner [19,20]. Diverse nuclear receptors, such as PPARα, PPARγ, PPARδ, glucocorticoid receptor (GR), estrogen receptor (ER), and liver X receptor (LXR) are also directly or indirectly associated with adiponectin production during adipogenesis in hBM-MSCs [21,22]. When adiponectin production-enhancing compounds are discovered in phenotype-based methods, a molecular target identification study is followed in the conventional drug discovery process.

Natural products derived from microorganisms like fungi have been screened to discover novel drug candidate molecules [23,24]. Secondary metabolites of marine-derived fungi have drawn attention as a unique source of novel pharmacophores. Recently, we reported that terrelumamide A and terrelumamide B, the secondary metabolites isolated from marine-derived fungal species, *Aspergillus terreus*, were adiponectin production-enhancing compounds during adipogenesis in hBM-MSCs [25]. In addition to these lumazine peptide terrelumamides, other adiponectin production-enhancing compounds were identified from the secondary metabolites of marine-derived *Aspergillus terreus* (Supplementary Figure S1). Among the active compounds, butyrolactone I exhibited the most potent adiponectin production-enhancing activity. Butyrolactone I is an inhibitor of cyclin-dependent kinase (CDK)s including CDK1 and CDK5 [26,27,28]. The protein kinase CDK5 can phosphorylate the serine (Ser) 245 residue of PPARγ. The phosphorylated PPARγ at Ser245 is associated with the down-regulation of insulin-sensitizing adiponectin production [29,30]. Therefore, it is expected that butyrolactone I increases adiponectin production during adipogenesis in hBM-MSCs. Although the CDK5 inhibitor activity of butyrolactone I is relatively weaker than those of the other specific CDK5 inhibitors like roscovitine, butyrolactone I has the most potent adiponectin production-enhancing activity among the CDK5 inhibitors. In this study, target identification was performed to explain the potent adiponectin production-enhancing activity of butyrolactone I.

## 2. Materials and Methods

### 2.1. Chemicals and Reagents

Butyrolactone I and other compounds were provided by Dr. Jongheon Shin (Seoul National University, Seoul, Republic of Korea). The extraction and isolation were conducted as previously reported [25].

Butyrolactone I 

Appearance: Pale yellow amorphous solid, [α]15D +95 (*c* 1.0, EtOH), FT-IR (KBr, cm^−1^): 3179, 1763; ^1^H-NMR (DMSO-*d*_6_) *δ* = 10.52 (brs, 1H), 9.92 (s, 1H), 9.12 (s, 1H), 7.50 (d, *J* = 8 Hz, 2H aromatic H), 6.88 (d, *J* = 8 Hz, 2H aromatic H), 6.53 (d, *J* = 7.5 Hz, 1H), 6.47 (dd, *J* = 8 Hz, 2 Hz, 1H), 6.37 (m, 1H), 5.01 (t, *J* = 7.1 Hz, 1H), 3.74 (s, 3H), 3.36 (m, 2H), 3.00 (t, *J* = 7 Hz, 2H), 1.62 (s, 3H), 1.53 (s, 3H); ^13^C-NMR (DMSO-*d*_6_) *δ* = 169.8, 167.9, 157.8, 153.7, 138.0, 131.3, 130.8, 128.7, 128.3, 126.4, 123.1, 122.3, 121.0, 115.7, 114.0, 84.7, 53.4, 38.0, 27.5, 25.4, 17.4; HR-ESI-MS: *m*/*z*: [M + H]^+^: 425.1598; calc. for C_24_H_25_O_7_: *m*/*z*: [M + H]^+^: 425.1595

### 2.2. Cell Culture, Adipocyte Differentiation, Adiponectin Enzyme-Linked Immunosorbent Assay (ELISA), and Oil Red O Staining

The hBM-MSCs (Product No. PT-2501) were acquired from Lonza (Walkersville, MD, USA) and the culturing media consisted of Dulbecco Modified Eagle’s Medium (DMEM; glucose 1 g/L), 1% penicillin-streptomycin (Invitrogen, Carlsbad, CA, USA), GlutamaxTM (Invitrogen), and 10% fetal bovine serum (FBS). To promote adipogenic differentiation, the medium was changed to adipogenic differentiation media consisting of DMEM (glucose 4.5 g/L), 1% penicillin-streptomycin (Invitrogen), 10% fetal bovine serum (FBS), 0.5 mM 3-isobutyl-1-methylxanthine (IBMX), 0.5 μM dexamethasone, and 10 μg/mL insulin (IDX adipogenic condition). IBMX (Product No. I7018), dexamethasone (Product No. D8893), insulin (Product No. I2643), pioglitazone (Product No. E6910), aspirin (Product No. A2093), and glibenclamide (Product No. G0639) were acquired from Sigma-Aldrich (St. Louis, MO, USA). Roscovitine (Cat. No. 1332), and Ro-3306 (Cat. No. 4181) were acquired from Tocris Bioscience (Bristol, UK). A-674563 (Cat. No. S2670), and AT7519 (Cat. No. S1524) were acquired from Selleck Chemicals (Houston, TX). To quantify adiponectin level in the cell supernatants, a QuantikineTM immunoassay kit (R&D Systems, Minneapolis, MN, USA) was used. The level of adiponectin was quantified as described previously [31]. Oil red O staining was used for evaluating the lipid droplet formation during adipogenesis in hBM-MSCs as described previously [32,33]. The lipid droplet in hBM-MSCs was photographed using an Olympus IX71 inverted phase-microscope (Olympus Co., Tokyo, Japan).

### 2.3. Total RNA Isolation and Quantitiative Real-Time PCR

The total RNA samples in the hBM-MSCs inducing adipogenic differentiation were extracted using Trizol^TM^ (Invitrogen). All Q-TR-PCR was performed with an Applied Biosystems 7500 Real-Time PCR system (Applied Biosystems) according to previously described methods. The Quantitiative Real-Time PCR (q-RT-PCR) primer sets (Applied Biosystems, Foster City, CA, USA) were used to quantify the transcription levels of ADIPOQ (Cat. No. Hs00605917_m1) and PPARG (Cat. No. Hs00234592_m1). Human glyceraldehyde-3-phosphate dehydrogenase (GAPDH, Cat. No. 4333764F), a house-keeping gene, was used as a reference gene. The relative levels of gene expression were quantified using equations derived from a mathematical model developed by Pfaffl [34].

### 2.4. Nuclear Receptor Binding Assays

To identify the binding of butyrolactone I to the GR, PPAR subtype α, γ, and δ/β, the time-resolved fluorescence resonance energy transfer (TR-FRET)-based competitive receptor binding assay was performed using Lanthascreen^TM^ competitive binding assay kits (Invitrogen, Cat. No. A15901, PV4892, PV4893, and PV4894). To evaluate the receptor coactivation of liver X receptor, Lanthascreen^TM^ coactivator assay kits were used (Invitrogen, Cat. No. PV4658). CLARIOstar (BMG LABTECH, Ortenberg, Germany) was used for the TR-FRET assay measurements and the instrument settings were the same as previously described [3].

### 2.5. Cloning, Protein Expression, and Purification

PPARγ ligand-binding domain (LBD) (residues 195-477 in PPARγ1 numbering) was cloned into the expression vector pET-28b(+) (Novagen) between Nde1 and Xho1 restriction sites containing an N-terminal hexahistidine (His_6_) tag (MGSSHHHHHHSSGLVPRGSH) and a thrombin cleavage site. The recombinant plasmid was transformed into Rossetta2(DE3) *Escherichia Coli* strain. The cells were grown at 37 ℃ in Luria Broth media containing 30 μg/mL kanamycin and induced by 0.5 mM isopropyl 1-thio-β-d-galactopyranoside at an OD_600_ of 0.6 and then incubated for additional 20 h at 20 ℃. The cells were harvested by centrifugation at 6000× *g* for 10 min and lysed by sonication in buffer A (20 mM Tris-HCl pH 8.5, 150 mM NaCl, 5 mM imidazole, 10% glycerol, and 1 mM TCEP) containing 1 mM phenylmethanesulfonylfluoride. The lysates were centrifuged at 35,000× *g* for an hour and the supernatants were filtered with a 0.45 μm syringe filter device (Sartorius, Göttingen, Germany). For affinity chromatography, they were loaded onto 5 mL HiTrap chelating HP column (GE Healthcare, Chicago, IL, USA) that was charged with Ni^2+^ and equilibrated with buffer A. Upon eluting with linear gradient of buffer B (20 mM Tris-HCl pH 8.5, 150 mM NaCl, 300 mM imidazole, 10% glycerol, and 1 mM TCEP), PPARγ LBD was eluted at an imidazole concentration of 50–100 mM. After the eluted protein was desalted using HiPrep Desalting column 26/10 (GE Healthcare) to buffer C (20 mM Tris-HCl pH 8.5, 150 mM NaCl, 10% glycerol, and 1 mM TCEP), the protein was treated with thrombin (Sigma-Aldrich) for the cleavage of His_6_-tag at 1 unit/mg and incubated at 4 ℃ overnight. The His_6_-tag-cleaved PPARγ LBD was purified by passing through the Ni^2+^ charged HiTrap chelating HP column (GE Healthcare) to remove His_6_-tag or uncleaved His_6_-tagged target proteins, followed by gel filtration chromatography column, HiLoad 16/600 Superdex 200 pg (GE Healthcare), that was previously equilibrated with buffer C. For crystallization, the PPARγ LBD was concentrated to 15.5 mg/mL using an Amicon Ultra-15 Centrifugal Filter Unit (Merck Millipore, Darmstadt, Germany).

### 2.6. Crystallization

The ligand-free PPARγ LBD crystals were grown by the sitting-drop vapor diffusion method at 22 ℃ by mixing 0.5 μL each of the purified protein sample and a crystallization solution containing 1.4 M sodium citrate tribasic dihydrate (Hampton Research, Aliso Viejo, CA, USA) and 0.1 M HEPES pH 7.5. The crystals suitable for data collection were grown in the presence of micro-seeds that were made from the initial crystals using Seed Bead Kits (Hampton Research) according to the manufacturer’s instructions. The cubic-shaped crystals with a dimension of approximately 0.2 mm × 0.2 mm × 0.2 mm were obtained within a few days. For butyrolactone I-bound PPARγ LBD, butyrolactone I was completely dissolved in 100% DMSO at 100 mM concentration and was soaked into ligand-free PPARγ LBD crystals with 1:5 molar ratio containing 1% (*v*/*v*) DMSO for 2−3 days.

### 2.7. X-Ray Data Collection, Structure Determination, and Refinement

The X-ray diffraction data for ligand-free PPARγ LBD were collected at 100 K using a Pilatus 3 6M detector system (Dectris Ltd., Baden, Switzerland) in the BL-11C experimental station at Pohang Light Source, Korea. The X-ray diffraction data for butyrolactone I-bound PPARγ LBD were collected at 100 K using an Eiger 9M detector system (DECTRIS) in BL-5C experimental station at Pohang Light Source, Korea. All data were processed and scaled using *HKL*2000 [35]. The crystals of butyrolactone I-bound and ligand-free PPARγ LBD belonged to the space group of C2, with unit cell parameters of a = 93.62 Å, b = 61.12 Å, c = 119.76 Å and α = 90.00°, β = 103.64°, γ = 90.00°; and of a = 93.15 Å, b = 62.26 Å, c = 119.50 Å and α = 90°, β = 102.51°, γ = 90°, respectively.

Both the structures were determined by molecular replacement with the previously published PPARγ LBD structure (PDB ID: 3VSO) as a phasing model using *MolRep* [36]. The structures were refined by iterative manual buildings in *Coot* [37] and *Refmac* [38] in the CCP4 program suite. All refinement steps were monitored using an R_free_ value [39] based on the independent reflections and the reliability of refined models was evaluated using *MolProbity* [40]. The statistics of data collection and refinement are summarized in Table 1.

### 2.8. In vitro Kinase Assay

In vitro kinase assay was performed as previously described [41]. Briefly, 1 μg of the purified PPARγ LBD was incubated with butyrolactone I, pioglitazone, and roscovitine at various concentrations ranging from 0.08 to 10 μM in the assay buffer (25 mM Tris-HCl pH 7.5, 5 mM β-glycerophosphate, 2 mM dithiothreitol, 0.1 mM Na_3_VO_4_, 10 mM MgCl_2_) containing 25 μM ATP for 30 min at 30 °C. Then, active CDK5/p35 (Eurofins Scientific, Dundee, UK, Cat. No. 14-477) was added and incubated with PPARγ LBD for 30 min more at 30 °C. The phosphorylated PPARγ LBD by CDK5/p35 was detected by western blotting using an anti-CDK substrate antibody targeting phospho-Ser in a [K/R]-S-P-X-[K/R] motif (Cell Signaling Technology, Danvers, MA, USA, Cat. No. 9477) and an anti-PPARγ antibody targeting residues surrounding His466 of PPARγ (Cell Signaling Technology, Cat. No. 2443).

### 2.9. Luciferase Reporter Gene Assay

The HEK293T cells were cultured in DMEM supplemented with 10% fetal bovine serum and 1% *v/v* penicillin and streptomycin. The cells were grown to 80% confluency at 37 °C, under an atmosphere containing 5% CO_2_ and then were plated in 96-well plates (15,000 cells per well). For luciferase assays, the HEK293T cells were transfected with 50 ng pcDNA3-PPARγ WT, 70 ng PPRE-X3-TK-luc (1015; Bruce Spiegelman, Addgene, Cambridge, MA, USA), and 10 ng pRL-SV40 (Promega, Wallisellen, Switzerland) using Lipofectamine 2000 (Invitrogen, Carlsbad, CA, USA) according to the manufacturer’s instruction. The cells were incubated for 24 h and then treated with 10 μM, 1.25 μM, 156.26 nM, 19.53 nM, and 2.44 nM butyrolactone I or pioglitazone. The luciferase activity was determined 20 h later with the Dual-Glo^®^ Luciferase Assay System 2000 (Promega), according to the manufacturer’s instruction, and the obtained results were normalized with the *Renilla* luciferase signal obtained with the pRL-SV40 plasmid. The relative luciferase units of treated cells over DMSO-treated control cells were plotted.

### 2.10. Statistical Analyses

RStudio^®^ for Windows (RStudio Inc., Boston, MA, USA) was used for statistical analysis. The experimental data are represented as the means ± standard deviation (SD, *n* = 3). One-way analysis of variance (ANOVA) and Tukey’s post hoc tests were performed to analyze the data. The level of significance was set at * *p* ≤ 0.05 and ** *p* ≤ 0.01.

### 2.11. Accession Codes

The atomic coordinates and structure factors for ligand-free and butyrolactone I-bound PPARγ LBD structures were deposited to the Protein Data Bank under the accession codes 6L8B and 6L89, respectively.

## 3. Results

### 3.1. Adiponectin Production-Enhancing Activity of Butyrolactone I in hBM-MSCs Adipogenic Model

**Figure 1:**
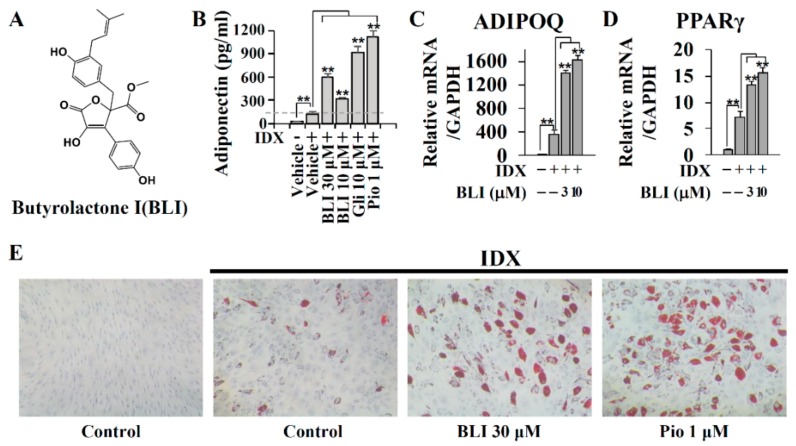
The adiponectin production-enhancing activity of butyrolactone I (BLI) during adipogenesis in human bone marrow-derived mesenchymal stem cells (hBM-MSCs). (**A**) The structure of butyrolactone I isolated from the marine-derived *Aspergillus terreus*. (**B**) Butyrolactone I was co-treated with the insulin (IDX) medium to induce adipogenesis in hBM-MSCs. On the fifth day of the adipogenic differentiation, cell culture supernatants were collected and the adiponectin production-enhancing activity was measured by ELISA. Glibenclamide (gli) and pioglitazone (pio) were used as positive controls. (**C** and **D**) In addition, on day 3, the total RNA was extracted and Q-RT-PCR was performed for adiponectin (*ADIPOQ*) and *PPARγ*. *GAPDH* was used as a reference control. (**E**) On the seventh day of adipogenic differentiation, ORO staining was performed to evaluate the lipid droplets formation. Values represent means ± standard deviation (SD, *n* = 3); * *p* ≤ 0.05, ** *p* ≤ 0.01.

Butyrolactone I has been reported to inhibit multiple CDKs, including CDK1 and CDK5 [42]. To confirm the inhibitory activity of butyrolactone I isolated from the marine fungus *A. terreus*, the CDK inhibition profile of butyrolactone I was determined against CDK1, CDK2, CDK3, CDK5, CDK6, CDK7, and CDK9 (Supplementary Figure S2). Butyrolactone I significantly inhibited the enzyme activity of CDK1/cyclin B, CDK2/cyclin A, CDK2/cyclin E, CDK5/p25, and CDK5/p35, whereas it did not significantly affect CDK3, CDK6, CDK7, or CDK9 (Supplementary Figure S2A). The half-maximum inhibition concentrations (IC_50_) of butyrolactone I for CDK1/cyclin B, CDK2/cyclin A, CDK2/cyclin E, CDK5/p25, and CDK5/p35 were 0.65, 1.38, 0.66, 0.17, and 0.22 μM, respectively (Supplementary Figure S2B). In general, CDK inhibitors inhibit cell cycle progression and induce the cellular apoptotic process [43,44]. At a non-cytotoxic concentration, butyrolactone I exhibited significant adiponectin production-enhancing activity as compared to the IDX control during adipogenesis in the hBM-MSCs (Figure 1A,B). The gene transcription of adiponectin and PPARγ was also upregulated following treatment with butyrolactone I in a concentration-dependent manner (Figure 1C,D). The nuclear receptor PPARγ is a pivotal transcription factor regulating mammalian adipogenesis and adipocyte functions [45]. The number and size of lipid droplets in the differentiated adipocytes were increased following treatment with butyrolactone I, although the effects were not as potent as those of pioglitazone, a clinically used PPARγ full agonist (Figure 1E).

### 3.2. The Adiponectin Production-Enhancing Activity of Butyrolactone I did not Correlate with the Potency of CDK5 Inhibition

**Figure 2:**
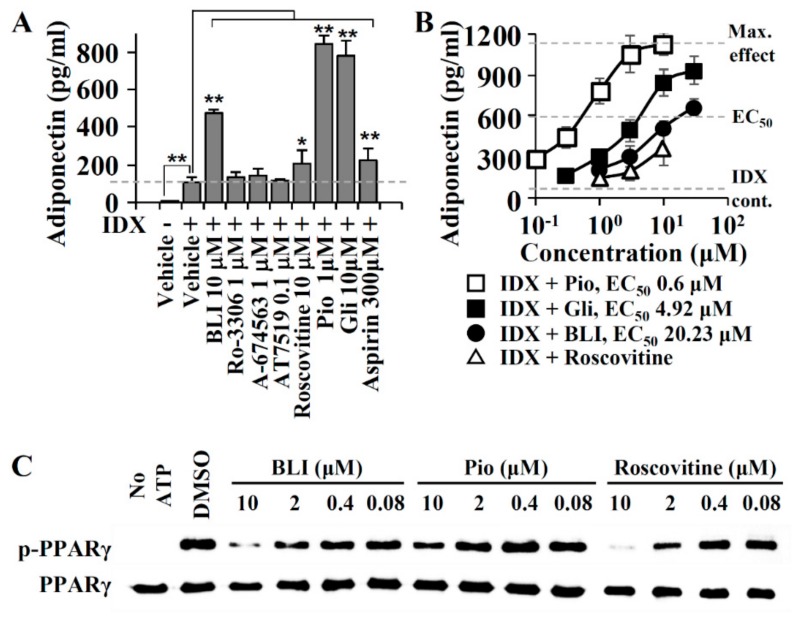
Effects of cyclin-dependent kinase (CDK5) inhibitors on adiponectin production during hBM-MSCs. (**A**) CDK inhibitors were added to IDX medium when adipogenic differentiation was induced in hBM-MSCs. On the fifth day, cell culture supernatants were collected and adiponectin level was quantified by ELISA. Pioglitazone (pio), glibenclamide (gli), and aspirin were used as positive controls for the adiponectin production-enhancing activity. Values represent means ± SD (*n* = 3); * *p* ≤ 0.05, ** *p* ≤ 0.01. (**B**) The half-maximal effective concentration (EC_50_) values were calculated for butyrolactone I and roscovitine on adiponectin production-enhancing activity. (**C**) Western blot analysis for measuring the inhibition activity against PPARγ phosphorylation (pPPARγ). PPARγ ligand-binding domain (LBD) was incubated with butyrolactone I (BLI), pioglitazone, or roscovitine at various concentrations in assay buffer containing ATP.

In obese and diabetic conditions, the phosphorylation of PPARγ by CDK5 is associated with a reduction in adiponectin expression [29]. Although CDK5 inhibitors have the potential to improve metabolic conditions associated with hypoadiponectinemia, the effects of CDK5 inhibitors on adiponectin biosynthesis has not been fully investigated. To compare the adiponectin production-enhancing activity of CDK5 inhibitors, hBM-MSCs were co-treated with butyrolactone I, Ro-3306, A-674563, AT7519, or roscovitine with IDX when adipogenesis was induced (Figure 2). Because CDK5 inhibitors are cytotoxic or apoptotic against diverse normal or cancer cell lines [46,47], the non-cytotoxic concentrations of Ro-3306, A-674563, AT7519, and roscovitine were chosen for screening their adiponectin production-enhancing activity (Figure 2A). As compared to that of the IDX control, butyrolactone I and roscovitine significantly enhanced adiponectin production, whereas the other CDK5 inhibitors Ro-3306, A-674563, and AT7519 exhibited no significant effects on adiponectin production in hBM-MSCs at non-cytotoxic concentrations (Figure 2A). In a concentration-response analysis, the half-maximal effective concentration (EC_50_) of butyrolactone I was 20.23 μM when the maximum effect of pioglitazone was set to the 100% maximum effect for the adiponectin production-enhancing activity (Figure 2B). Although roscovitine enhanced adiponectin production by 2.02-fold as compared to that of the IDX control, roscovitine was below the half-maximal level of pioglitazone at a non-cytotoxic concentration (Figure 2B). In adipocytes, the PPARγ agonists significantly decreased the Ser245 phosphorylation of PPARγ [29]. To determine whether the inhibition of PPARγ phosphorylation was correlated with the adiponectin production-enhancing activity, the Ser245 phosphorylation levels in each sample treated with butyrolactone I, roscovitine, or pioglitazone were compared (Figure 2C). In Western blotting for the phospho-Ser245 in PPARγ, butyrolactone I inhibited PPARγ phosphorylation in a concentration-dependent manner, which was as potent as roscovitine. Although pioglitazone inhibited Ser245 phosphorylation, its activity was far as lower compared to that of butyrolactone I (Figure 2C). Therefore, the inhibitory activity of butyrolactone I against the PPARγ phosphorylation did not correlated with the adiponectin production-enhancing activity. In this regard, it was possible that the relatively potent adiponectin production-enhancing activity of butyrolactone I can be achieved by the other pharmacological mechanisms, as well as by CDK5 inhibition.

### 3.3. A PPARγ Partial Agonist Activity of Butyrolactone I

**Figure 3:**
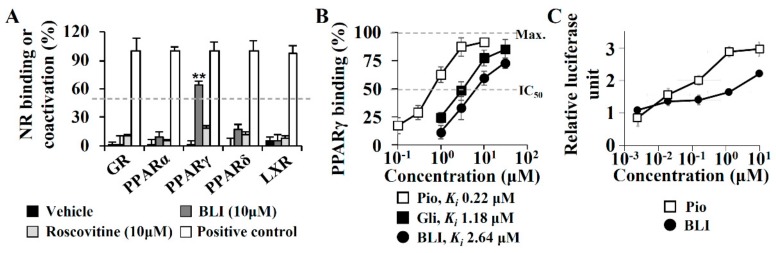
Molecular target identification of butyrolactone I. (**A**) Competitive time-resolved fluorescence resonance energy transfer (TR-FRET)-based nuclear receptor (glucocorticoid receptor (GR), PPAR subtype α/γ/δ, and iver X receptor (LXR)) binding assay was performed for butyrolactone I and roscovitine. (**B**) *Ki* values for PPARγ binding were calculated by the Cheng and Prusoff equation. Pioglitazone (pio) and glibenclamide (gli) were used as positive controls. (**C**) Cell-based PPARγ luciferase reporter transactivation assay was performed. HEK293T cell was co-transfected with an expression vector encoding full length PPARγ, a reporter vector encoding the firefly luciferase following PPARγ response element (PPRE), and a normalization vector encoding the *Renila* luciferase. After the treatment of the compounds including the butyrolactone I for 20 h to the cells, the signals of luminescence were detected and normalized. Values represent means ± SD (*n* = 3); * *p* ≤ 0.05, ** *p* ≤ 0.01. IC_50_: half-maximum inhibition concentrations.

The target identification of butyrolactone I was performed due to a lack of correlation between the adiponectin production-enhancing activities and CDK5 inhibitor efficacies. Adiponectin biosynthesis is regulated by nuclear receptors, such as PPARα, PPARγ, PPARδ, GR, ER, and LXR [21,22]. To determine whether butyrolactone I directly affected these nuclear receptors, time-resolved fluorescence resonance energy transfer (TR-FRET)-based nuclear receptor binding assays were performed (Figure 3). In a TR-FRET assay, butyrolactone I significantly bound to PPARγ but did not affect specific ligand binding to PPARα, PPARδ, GR, or LXR. In contrast, roscovitine did not replace ligand binding to any nuclear receptor evaluated in this study (Figure 3A). In a concentration-dependent analysis of PPARγ binding, the *Ki* value of butyrolactone I was 2.64 μM, which was lower than those of a PPARγ full agonist pioglitazone (*Ki* 0.22 μM) and a PPARγ-binding sulfonylurea antidiabetic glibenclamide (*Ki* 1.18 μM) (Figure 3B). To determine the PPARγ binding activity of butyrolactone I with its functional association, butyrolactone I-induced PPARγ transactivation was measured with a cell-based PPARγ luciferase reporter transactivation assay. Butyrolactone I significantly increased PPARγ transactivation in a concentration-dependent manner; however, its maximum activity was not as potent as that of pioglitazone, supporting that butyrolactone I may be a PPARγ partial agonist (Figure 3C).

### 3.4. The Crystal Structure of PPARγ LBD in Complex with Butyrolactone I

**Figure 4:**
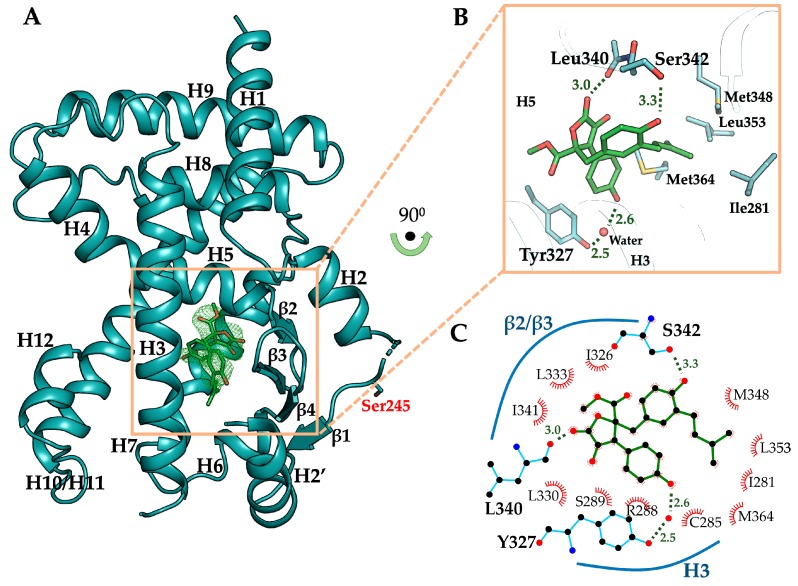
Overall structure of butyrolactone I-bound PPARγ LBD and its binding mode. (**A**) Butyrolactone I-bound PPARγ LBD (chain B) displayed as cartoon representation colored in cyan. Ser245, a CDK5-mediated phosphorylation site, is shown as a stick model. Butyrolactone I occupying the ligand binding pocket is shown as green-colored stick model. The omit map (*mFo–DFc*, contoured at 2.0σ) of butyrolactone I is displayed in green-colored mesh representation. (**B**) Close-up view of the ligand binding pocket. Butyrolactone I shown as green-colored stick model occupies the ligand binding pocket forming three hydrogen bonds with PPARγ LBD residues Leu340, Ser342, and Tyr327 (shown as stick models), including a water-mediated hydrogen bond. The hydrogen bond-mediating water molecule is shown as red sphere. Residues involved in the interaction with 3-methyl-2-butenyl group of butyrolactone I are shown as stick models (Ile281, Met348, Leu353, and Met364 in the hydrophobic pocket of PPARγ LBD). (**C**) PPARγ-butyrolactone I interactions of the crystal structure were analyzed using LigPlot+ and presented in a two-dimensional scheme.

To elucidate the binding mode of butyrolactone I to PPARγ, the crystal structure of PPARγ LBD in complex with butyrolactone I was determined at 2.1 Å resolution (Table 1). The crystal structure of butyrolactone I-bound PPARγ LBD adopted the canonical nuclear receptor fold consisting of three layers of 13 α-helices and 4 stranded β-sheet (Figure 4A). There were two protein molecules in an asymmetric unit, which were nearly identical to the previously published PPARγ LBD structure (Protein Data Bank (PDB) ID: 1PRG) with a root-mean-square deviation (RMSD) of 0.66 Å over 503 equivalent C_α_ atoms [48]. The ligand omit map calculated from the refined model clearly showed electron densities to model butyrolactone I (Figure 4A, Supplementary Figure S3). Butyrolactone I was bound to the canonical ligand-binding pocket (LBP) via three hydrogen bonds and hydrophobic effects (Figure 4B). The oxygen atom of the benzyl group and the oxygen atom of the lactone group in butyrolactone I formed hydrogen bonds with O_γ_ atom of Ser342 (hydrogen bond distance 3.3 Å) and with the backbone oxygen atoms of Leu340 (hydrogen bond distance 3.0 Å), respectively. The oxygen atom of hydroxylphenyl group in butyrolactone I participated in a water-mediated hydrogen bond with O_η_ atom of Tyr327 (hydrogen bond distances 2.6 Å and 2.5 Å). The hydrophobic effects of butyrolactone I were presented mostly with H3, H5, and β-sheet of PPARγ LBD, including Ile281, Cys285, Arg288, Ser289, Ala292, Ile326, Leu330, Leu333, Ile341, and Met364 (Figure 4C). In particular, the 3-methyl-2-butenyl group of butyrolactone I was surrounded by hydrophobic residues, including Ile281, Met348, Leu353, and Met364, which formed a hydrophobic pocket (Figure 4B). On the contrary, butyrolactone I did not directly interact with the O_η_ atom of Tyr473 on H12 that is known for the major interactive motif of the PPARγ full agonist in PPARγ LBD [48].

**Figure 5:**
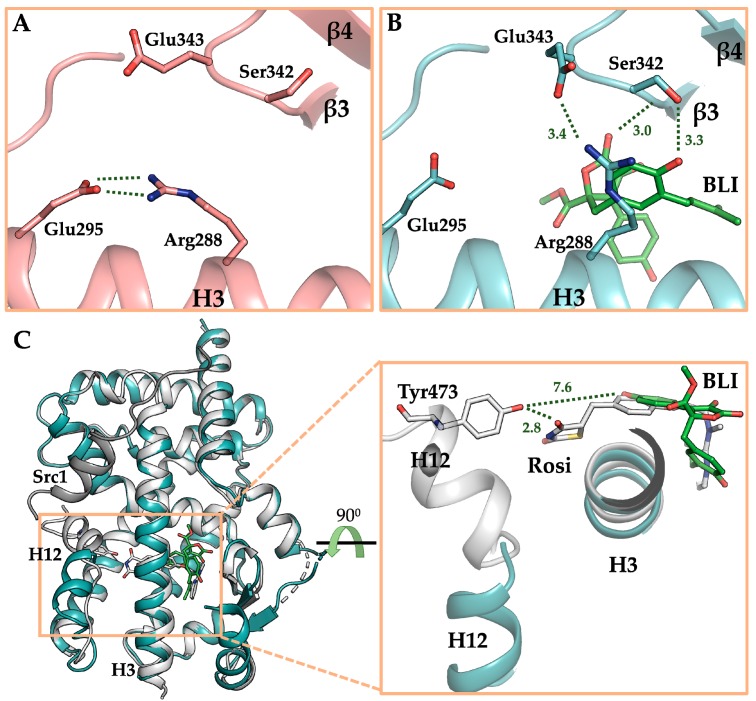
Binding mode of butyrolactone I to PPARγ LBD. (**A**) Ligand binding pocket (LBP) of ligand-free PPARγ LBD. (**B**) LBP of butyrolactone I-bound PPARγ LBD. The structures of ligand-free PPARγ LBD and butyrolactone I-bound PPARγ LBD are displayed as cartoon representations in salmon and cyan, respectively. Butyrolactone I shown as green-colored stick model occupied LBP. Residues inducing conformational changes upon binding of butyrolactone I to PPARγ LBD (Arg288, Ser342, and Glu343) are shown as stick models and hydrogen bonding networks are depicted by green-dashed lines. (**C**) Comparison of the structures of rosiglitazone (Rosi)-bound PPARγ LBD and butyrolactone I-bound PPARγ LBD. The structures of rosiglitazone-bound and butyrolactone I-bound PPARγ LBD are superposed and shown as cartoon representations in white and cyan, respectively. The structure of rosiglitazone-bound PPARγ LBD is shown in its active conformation in the presence of co-activator Src1 colored in dark grey. In the close-up view of PPARγ LBP, rosiglitazone and butyrolactone I are displayed as stick models in white and green, respectively. Tyr473 on H12 is displayed as a stick model and distances between Tyr473 and rosiglitazone or butyrolactone I are depicted by dashed lines.

For a precise comparison, the crystal structure of ligand-free PPARγ LBD under the same condition with butyrolactone I-bound PPARγ LBD was also determined at 2.1 Å resolution. Both the structures were determined in the same monoclinic space group *C*2 with nearly identical unit cell parameters and were similar to each other with RMSD of 0.53 Å over 513 equivalent C_α_ atoms (Supplementary Figure S4). Compared to the ligand-free PPARγ LBD structure, binding of butyrolactone I to PPARγ LBD induced conformational changes of Ser342 and Glu343 on the β-sheet in that they formed hydrogen bonds with the oxygen atom of benzyl group in butyrolactone I and Arg288 of PPARγ LBD, respectively (Figure 5A,B). In particular, Arg288 was observed in the alternative conformation, forming a hydrogen bond with Glu343. In addition to the hydrogen bond between butyrolactone I and Leu340, three new hydrogen bonds were formed in the butyrolactone I-bound structure with the residues on the β-sheet, contributing to its structural stabilization.

In the crystal structure of the butyrolactone I-bound PPARγ LBD, the binding mode of butyrolactone I was similar to those of the well-known PPARγ partial agonists [49], which was consistent with the results of the butyrolactone I-induced PPARγ transactivation assay. To elucidate the structural differences between butyrolactone I and full agonists, we superposed the structure of butyrolactone I-bound PPARγ LBD to the structure of rosiglitazone-bound PPARγ LBD showing an active form with co-activator (Figure 5C). While the thiazolidinedione moiety of rosiglitazone formed a hydrogen bond with the O_η_ atom of Tyr473 at a distance of 2.8 Å, butyrolactone I did not interact with Tyr473 since the distance between the oxygen atom of hydroxylphenyl group in butyrolactone I and the O_η_ atom of Tyr473 was 7.6 Å. Instead, butyrolactone I mainly interacted with H3 and the β-sheet in PPARγ LBD as shown with previously reported partial agonists. Taken together, both the PPARγ transactivation assay and the butyrolactone I-bound crystal structure suggest that butyrolactone I exhibited a partial agonism when bound to PPARγ.

## 4. Discussion

Butyrolactone I is a poly-CDK modulator significantly inhibiting CDK1 and CDK5 and has been considered as an anti-cancer drug candidate [43,44,50]. In phenotypic screening for adiponectin-production enhancing compounds with therapeutic potentials in various metabolic diseases, butyrolactone I significantly enhanced adiponectin production in non-cytotoxic concentrations. Due to the role of the CDK5-mediated Ser245 phosphorylation in the regulation of PPARγ functions, the adiponectin production-enhancing activity of butyrolactone I was expected. However, the adiponectin production-enhancing activity of butyrolactone I was not correlated with its CDK5 inhibitor potency when compared with those of other adiponectin production-enhancing CDK5 inhibitors, such as roscovitine. As a result, it was expected that butyrolactone I can affect other cellular targets associated with adiponectin production during adipogenesis in the hBM-MSCs. In a target identification study, we demonstrated that the adiponectin production-enhancing activity of butyrolactone I was significantly mediated by its PPARγ partial agonist activity. The additive or synergistic potency can be explained by the concept of polypharmacology to explain the pleiotropic or potent activities of multiple-targeting drugs [51,52]. Therefore, the potent adiponectin production-enhancing activity of butyrolactone I can be explained with its polypharmacophore associated with both the inhibition of CDK5 and the transactivation of PPARγ.

CDK5 regulates cell cycle progression, cellular proliferation, and cell migration, and therefore, dysregulation of CDK5 in cellular functions can cause oncogenesis leading to lung and colorectal cancer [46,47]. Notably, when the cancer cells were treated with inhibitors of PPARγ phosphorylation, the cancer cells showed more susceptibility to anti-cancer agents inducing DNA damage responses [53]. In addition, CDK5 plays a role in the pathogenesis of various metabolic diseases. When obesity was induced by a high-fat diet, CDK5 and its associated PPARγ phosphorylation were upregulated in vivo, which led to impaired insulin sensitivity [29]. PPARγ ligands are prescribed and have been studied to improve metabolic pathologic outcomes in type II diabetes, dyslipidemia, and nonalcoholic steatohepatitis [54,55]. PPARγ, a pivotal nuclear receptor regulating cellular metabolic processes, is also expressed in various carcinomas including colorectal, breast, pancreatic, and lung cancers. The transactivation of PPARγ induces apoptosis and inhibits proliferation in human cancer cell lines [56]. In fact, the use of PPARγ agonists in human cancer therapeutics has been studied [57]. Because the polypharmacological effects of butyrolactone I on both CDK5 and PPARγ can offer new therapeutic opportunities to treat metabolic diseases and cancers, further studies should be directed to elucidate the polypharmacological outcome of butyrolactone I in various in vivo disease models, especially associated with its additive or synergistic effects on both metabolic diseases and human cancers.

Butyrolactone I functions as a PPARγ partial agonist. Currently, drug discovery programs targeting PPARγ have tried to design PPARγ partial modulators because PPARγ full agonists have serious adverse effects, such as fluid retention, congestive heart failure, bone fractures, and hepatotoxicity [58]. In the crystal structure of the butyrolactone I-bound PPARγ LBD, butyrolactone I bound to the hydrophobic binding pocket consisted of H3, H5, and β-sheet, which is a commonly observed binding mode for PPARγ partial agonists [49]. PPARγ full agonists, such as rosiglitazone and pioglitazone, interact via hydrogen bonds with Tyr473 of PPARγ which is essential for the stabilization of H12, resulting in the PPARγ coactivator protein binding [48]. In contrast, butyrolactone I did not interact with Tyr473 in H12 and instead it formed hydrogen bonds with Ser342 and Glu343 on the β-sheet and occupied the hydrophobic pocket, which is a typical feature of the PPARγ partial agonists [49]. In addition, both the β-sheet stabilization via interaction of Ser342 with ligands and the ligand binding to the hydrophobic pocket of PPARγ LBD have been proposed as inhibitory mechanisms of CDK5-mediated PPARγ Ser245 phosphorylation [59,60]. In our previous studies, we also reported that the binding of imatinib and SB1495 to the hydrophobic pocket inhibits CDK5-mediated Ser245 phosphorylation [61,62]. Consistent with previous studies, Ser245 phosphorylation inhibition by butyrolactone I could be attributed to the binding of butyrolactone I to the hydrophobic pocket and interacting with several residues on the β-sheet, including Ser342. It is interesting that butyrolactone I acts as a CDK5 inhibitor and inhibits CDK5-mediated PPARγ phosphorylation simultaneously. Besides, the chemical structure of butyrolactone I is different from those of other known PPARγ partial agonists [63]. Therefore, butyrolactone I provides a new polypharmacophore to design novel PPARγ partial agonists having CDK inhibitor activities.

## 5. Conclusions

In the phenotype screening to find novel adiponectin production-enhancing compounds and the following target identification, butyrolactone I purified from the marine fungus *A. terreus* was demonstrated to be a polypharmacological compound simultaneously targeting CDKs and PPARγ. The pathologic changes of CDK5 and PPARγ in a various metabolic diseases and cancer have been well addressed. Therefore, butyrolactone I has additive or synergistic therapeutic potentials in diseases with multi-factorial etiologies. In addition, the polypharmacophore of butyrolactone I and the crystal structure complexed with PPARγ LBD provide better opportunities to design a novel PPARγ partial agonist expecting therapeutic synergism.

## Figures and Tables

**Table 1:** Statistics for the data collection and model refinement.

Model Name	Butyrolactone I-Bound PPARγ LBD	Ligand-Free PPARγ LBD
Data collection		
X-ray source	PLS-5C	PLS-11C
X-ray wavelength (Å)	1.0000	0.9794
Space group	*C*2	*C*2
Unit cell parameters		
a, b, c (Å)	93.62, 61.12, 119.76	93.15, 62.26, 119.50
α, β, γ (⁰)	90.00, 103.64, 90.00	90.00, 102.51, 90.00
Resolution range (Å)	50.00–2.10 (2.14–2.10)^a^	30.00–2.10 (2.14–2.10)^a^
Total/unique reflections	177,451/38,329	174,642/38,322
Redundancy	4.6 (4.4)	4.6 (4.5)
Completeness (%)	99.3	98.0
<I/σ_I_>	39.3 (2.0)^a^	12.6 (2.3)^a^
*R_merge_^b^*(%)	3.2 (71.0)^a^	10.1 (58.3)^a^
CC_1/2_	1.00 (0.81)^a^	0.99 (0.82)^a^
Model refinement		
Resolution range (Å)	50.00–2.10	30.00–2.10
*R_work_*/*R_free_*^c^ (%)	22.8/26.8	21.8/26.8
No. of non-hydrogen atoms		
Protein	4129	4230
Ligand (butyrolactone I)	62	-
water	150	159
Average B factor (Å^2^)		
Protein	35.4	31.5
Ligand (butyrolactone I)	68.7	-
Water	31.2	28.3
R.m.s. deviations from ideal geometry		
Bond lengths (Å)	0.0042	0.0034
Bond angles (⁰)	1.2587	1.2024
Ramachandran plot^d^		
Favored/Outliers (%)	98.4/0	98.6/0
Poor rotamers^d^ (%)	0	0

^a^ Values in parentheses refer to the highest resolution shell. ^b^*R_merge_* = Σ_h_Σ_i_|I(h)_i_–<I(h)>|/Σ_h_Σ_i_I(h)_i_, where I(h) is the intensity of reflection h, Σ_h_ is the sum over all reflections, and Σ_i_ is the sum over i measurements of reflection h. *^C^ R_free_* = Σ||*F*_obs_|–|*F*_calc_||/Σ|*F*_obs_|, where *R*_free_ is calculated for a randomly chosen 5% of reflections that were not used for structure refinement. *R*_work_ is calculated for the remaining reflections. ^d^ Values obtained using *MolProbity*. LBD: ligand-binding domain; PPARγ: peroxisome proliferator-activated receptor γ; R.m.s.: root mean square.

## Supplementary Materials

The following are available online at https://www.mdpi.com/2218-273X/10/2/275/s1, Figure S1: Adiponectin production-enhancing activity of secondary metabolite of the marine-derived *Aspergillus terreus*. Figure S2: The kinase inhibitor assay with butyrolactone I. Figure S3: Electron density maps of butyrolactone I in chain B of butyrolactone I-bound PPARγ LBD. Figure S4: Structural comparison between ligand-free and butyrolactone I-bound PPARγ LBD. Figure S5: The ^1^H NMR (600 MHz, DMSO-*d*_6_) spectrum of butyrolactone I. Figure S6: The ^13^C NMR (150 MHz, DMSO-*d*_6_) spectrum of butyrolactone I. Full length blots used in Figure 2.
