# Supplementary material for: Cyclin-Dependent Kinase 5 Inhibitor Butyrolactone I Elicits a Partial Agonist Activity of Peroxisome Proliferator-Activated Receptor γ
Source: Biomolecules. 2020 Feb 11;10(2):275. doi: 10.3390/biom10020275 (PMC7072624; doi:10.3390/biom10020275)
Supplement: Supplementary file 1 [file biomolecules-10-00275-s001.pdf]

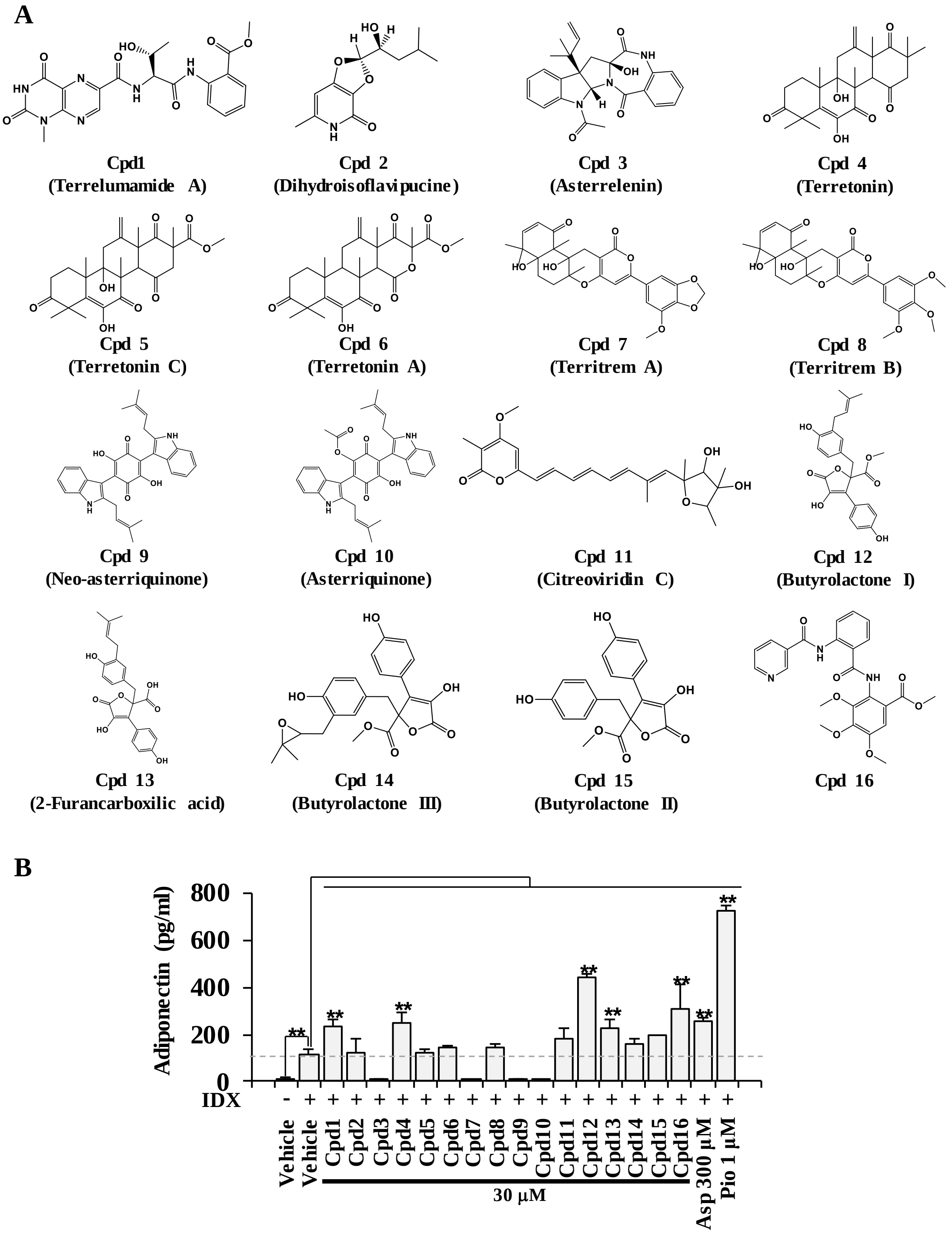

Figure S1. Adiponectin production-enhancing activity of secondary metabolite of the marine-derived *Aspergillus terreus*. (A) The structure of secondary metabolite of the marine-derived *Aspergillus terreus*. (B) Secondary metabolite were added to IDX medium when adipogenic differentiation was induced in hBM-MSCs. Cell culture supernatants were collected and adiponectin level was measured by ELISA on the fifth day. Pioglitazone (pio), and aspirin were used as positive controls. Values represent means ± SD ($n = 3$); * $p \leq 0.05$, ** $p \leq 0.01$.

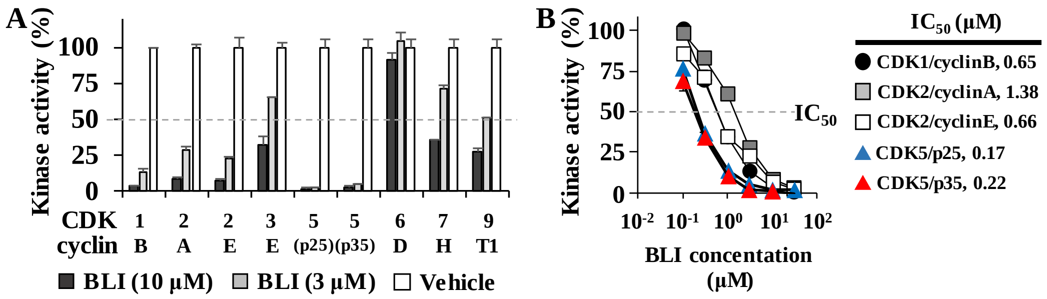

Figure S2. The kinase inhibitor assay with butyrolactone I. (A) The kinase activity was evaluated by measuring the γ-^{32}P-ATP incorporation to CDK complexes. The inhibitory activities of butyrolactone I on the phosphorylation of CDK1/cyclin B, CDK2/cyclin A, CDK2/cyclin E, CDK3/cyclin E, CDK5/p25, CDK5/p35, CDK6/cyclin D, CDK7/cyclin H, and CDK9/cyclin T1 were tested at each K_m ATP concentration. DMSO was included in each negative control. (B) The IC_{50} values were calculated for butyrolactone I on kinase inhibition activity. Values were expressed in terms of percentage compared to each positive control.

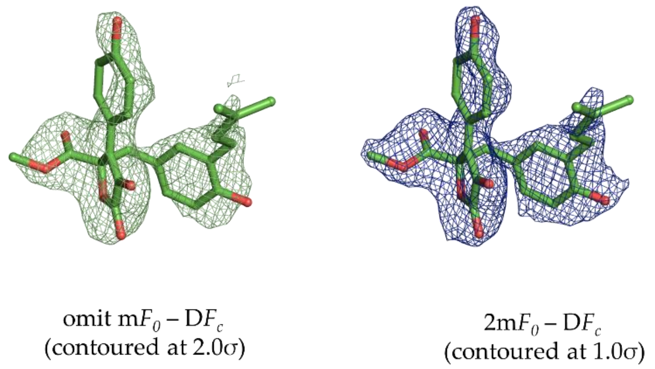

Figure S3. Electron density maps of butyrolactone I in chain B of butyrolactone I-bound PPARγ LBD. An omit map (mFo–DFc, contoured at 2.0σ) of butyrolactone I is displayed in green-colored mesh representation (left) and a model-refined map (2mFo–DFc, contoured at 1.0σ) of butyrolactone I is displayed in blue-colored mesh representation (right).

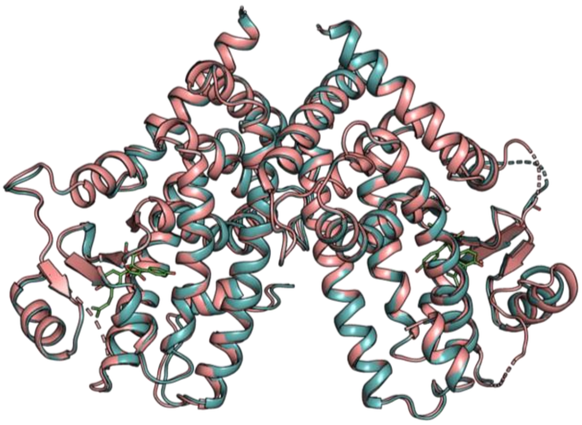

Figure S4. Structural comparison between ligand-free and butyrolactone I-bound PPARγ LBD. The structures of ligand-free PPARγ and butyrolactone I-bound PPARγ are displayed as cartoon representations in salmon and cyan, respectively. There are two PPARγ LBD molecules in an asymmetric unit (chain A and chain B). Two butyrolactone I molecules are shown as green-colored stick models and bound to the ligand binding pockets of PPARγ LBD.

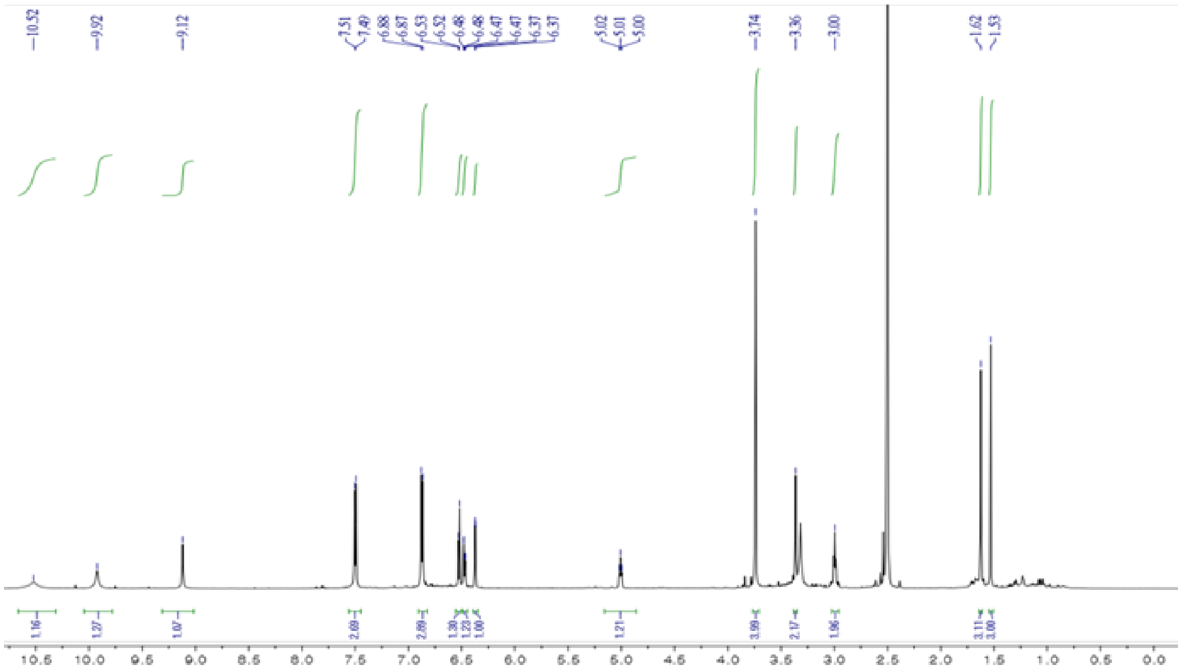

Figure S5. The ^{1}H NMR (600 MHz, DMSO-d_6) spectrum of butyrolactone I

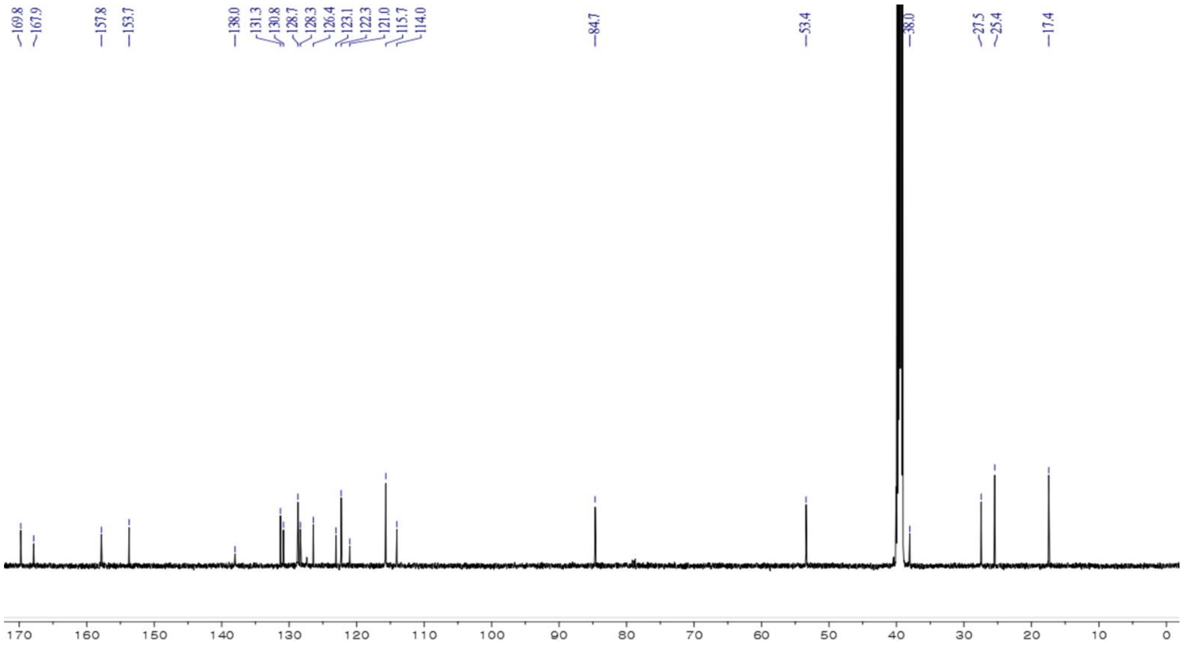

Figure S6. The ^{13}C NMR (150 MHz, DMSO-d_6) spectrum of butyrolactone I

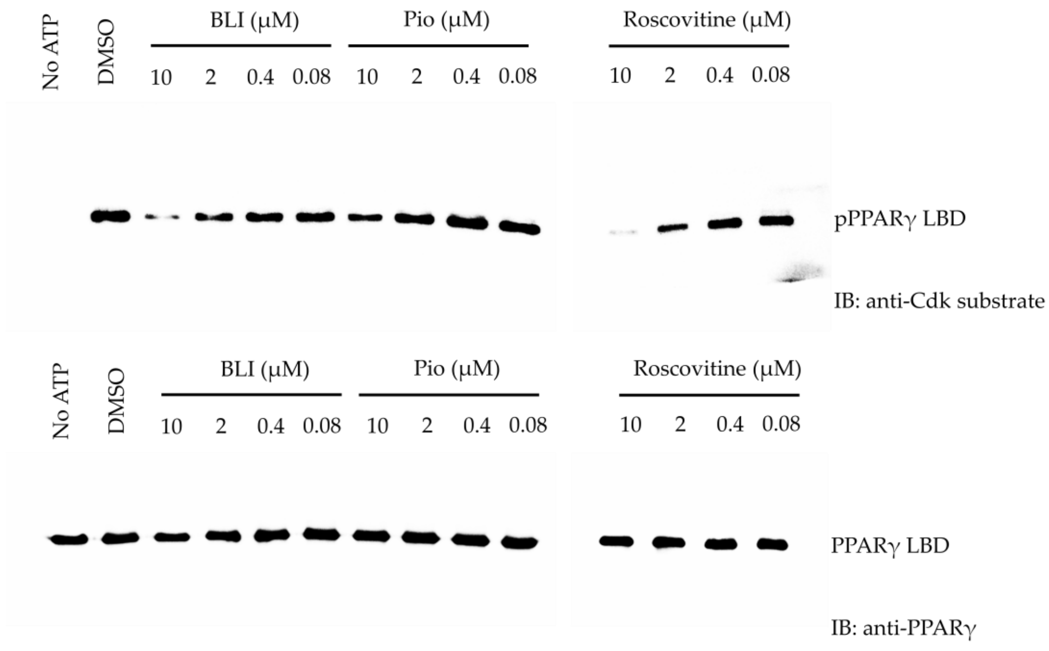

[Full length blots used in Figure 2 in the main manuscript.]
